# Complete Genome Sequence of “*Candidatus* Phytoplasma asteris” RP166, a Plant Pathogen Associated with Rapeseed Phyllody Disease in Poland

**DOI:** 10.1128/MRA.00760-20

**Published:** 2020-08-27

**Authors:** Shu-Ting Cho, Agnieszka Zwolińska, Weijie Huang, Roland H. M. Wouters, Sam T. Mugford, Saskia A. Hogenhout, Chih-Horng Kuo

**Affiliations:** a Institute of Plant and Microbial Biology, Academia Sinica, Taipei, Taiwan; b Virology and Bacteriology Department, Institute of Plant Protection, National Research Institute, Poznań, Poland; c Department of Crop Genetics, John Innes Centre, Norwich Research Park, Norwich, Norfolk, United Kingdom; University of Maryland School of Medicine

## Abstract

The complete genome sequence of “*Candidatus* Phytoplasma asteris” RP166, which consists of one 829,546-bp circular chromosome, is presented in this work. This bacterium is associated with rapeseed phyllody disease in Poland and belongs to the 16SrI-B (i.e., aster yellows) group.

## ANNOUNCEMENT

Phytoplasmas are plant pathogens transmitted by phloem-feeding insects from the order *Hemiptera*. Rapeseed (*Brassica napus* L.) crops are persistently threatened by “*Candidatus* Phytoplasma asteris” (aster yellows group, 16SrI-B subgroup), which causes phyllody diseases (1, 2). Phyllodies are leafy structures that develop in place of flowers and transform the infected plants into sterile “zombies” (i.e., plants that serve only for phytoplasma propagation). These dramatic morphological changes are induced by bacterial effectors (3). To better study the epidemiology of this disease, genomic resources are in great demand for the investigation of pathogenicity genes and the development of molecular markers.

Strain RP166 was collected from a naturally infected winter rapeseed plant at the Field Experimental Station of the Institute of Plant Protection–National Research Institute (Winna Góra, Poland; coordinates, 52.208921, 17.437842). Healthy Macrosteles laevis leafhoppers (*Cicadellidae*) were allowed to feed on the infected plant to acquire the bacteria. Next, the insects were maintained on healthy barley for several weeks to increase the phytoplasma titers prior to DNA extraction.

Two platforms were used for shotgun sequencing. For Illumina, a cetyltrimethylammonium bromide (CTAB) buffer protocol (4) was used for DNA extraction, followed by the use of a KAPA library preparation kit (catalog number KK8234) and Invitrogen SizeSelect gels (catalog number G6610-02) for ∼600-bp fragments. The MiSeq 2 × 300-bp paired-end sequencing (v3 chemistry) produced ∼10 Gb raw reads. For Oxford Nanopore Technologies (ONT), DNA was prepared using the Illustra Nucleon Phytopure kit (catalog number RPN8510) according to Wouters et al. (5). The library was prepared using the ONT ligation kit (catalog number SQK-LSK109) without shearing or size selection. The MinION run (R9.4 chemistry) produced 1,697,567 raw reads (∼5.9 Gb; *N*_50_, 12,607 bp). Guppy v2.3.1 was used for base calling with a minimum quality score of 7; no further processing of the ONT reads was conducted.

The analysis procedure was modified from those described in our previous studies (6, 7). The Illumina reads were quality (Q) trimmed with a Q20 cutoff; reads shorter than 100 bp were discarded. *De novo* assembly was performed using Velvet v1.2.10 (8) (parameters: hash_length=91, scaffolding=no, exp_cov=30, cov_cutoff=5, max_coverage=500, min_contig_lgth=2000). Putative phytoplasma contigs were identified based on BLASTX (9) searches against a custom database of protein sequences from available “*Ca*. Phytoplasma asteris” genomes (10). Then, the ONT reads were mapped to the contigs using minimap2 v2.15 (11) to produce a circular scaffold. Finally, an iterative process was used until the assembly was completed. In each iteration, the Illumina reads were mapped using BWA v0.7.12 (12), checked using SAMtools v1.2 (13), and inspected using IGV v2.3.57 (14). For gene prediction, RNAmmer (15), tRNAscan-SE (16), and Prodigal (17) were used. The annotation was based on the homologs in other phytoplasmas (10), as identified by OrthoMCL (18), followed by manual curation using BlastKOALA (19) and GenBank (20). The chromosome was rotated to have *dnaA* as the first gene.

Strain RP166 has one 829,546-bp circular chromosome with 27.7% G+C content; no plasmids were found. The Illumina and ONT reads provided 116× and 597× coverage, respectively. The annotation contains 6 rRNA genes, 32 tRNA genes, 753 protein-coding genes, and 69 pseudogenes.

### Data availability.

The raw reads have been deposited at the NCBI Sequence Read Archive under the accession numbers SRR12000858 and SRR12000859. The genome sequence has been deposited at GenBank/ENA/DDBJ under the accession number CP055264.
